# (*E*)-2-Fluoro-*N*′-(4-nitro­benzyl­idene)benzo­hydrazide

**DOI:** 10.1107/S1600536810052268

**Published:** 2010-12-18

**Authors:** Hong-Yun Wu, Hong-Yan Ban, Jia-Bo Wang, Li-Hua Zhang

**Affiliations:** a School of Chemical Engineering, University of Science and Technology Liaoning, Anshan 114051, People’s Republic of China

## Abstract

In the title hydrazone compound, C_14_H_10_FN_3_O_3_, the dihedral angle between the two substituted benzene rings is 13.7 (3)°. The mol­ecule exists in a *trans* configuration with respect to the central methyl­idene unit. In the crystal, mol­ecules are linked through inter­molecular N—H⋯O hydrogen bonds, forming chains along the *a* axis.

## Related literature

For the biological activity of hydrazones, see: Zhong *et al.* (2007 ▶); Raj *et al.* (2007 ▶); Jimenez-Pulido *et al.* (2008 ▶). For related structures, see: Ban (2010 ▶); Ban & Li (2008*a* ▶,*b* ▶); Li & Ban (2009*a* ▶,*b* ▶); Yehye *et al.* (2008 ▶); Fun, Patil, Jebas *et al.* (2008 ▶); Fun, Patil, Rao *et al.* (2008 ▶); Yang *et al.* (2008 ▶); Ejsmont *et al.* (2008 ▶).
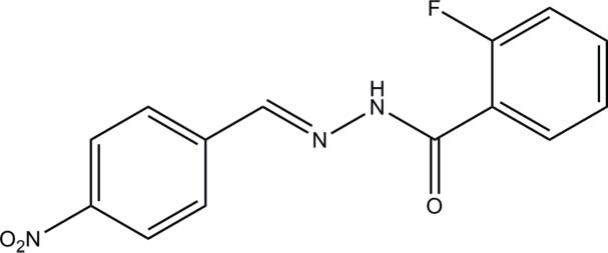


## Experimental

### 

#### Crystal data

C_14_H_10_FN_3_O_3_
*M* *_r_* = 287.25
Monoclinic, 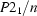

*a* = 7.077 (2) Å
*b* = 25.718 (4) Å
*c* = 7.6844 (17) Å
β = 111.640 (3)°
*V* = 1300.1 (5) Å^3^
*Z* = 4
Mo *K*α radiation
μ = 0.12 mm^−1^
*T* = 298 K
0.17 × 0.15 × 0.15 mm

#### Data collection

Bruker SMART CCD area-detector diffractometer
Absorption correction: multi-scan (*SADABS*; Sheldrick, 1996 ▶) *T*_min_ = 0.981, *T*_max_ = 0.983
6999 measured reflections
2810 independent reflections
1155 reflections with *I* > 2σ(*I*)
*R*_int_ = 0.074

#### Refinement

*R*[*F* ^2^ > 2σ(*F* ^2^)] = 0.067
*wR*(*F* ^2^) = 0.173
*S* = 0.98
2810 reflections
193 parameters
1 restraint
H atoms treated by a mixture of independent and constrained refinement
Δρ_max_ = 0.25 e Å^−3^
Δρ_min_ = −0.22 e Å^−3^

### 

Data collection: *SMART* (Bruker, 1998 ▶); cell refinement: *SAINT* (Bruker, 1998 ▶); data reduction: *SAINT*; program(s) used to solve structure: *SHELXS97* (Sheldrick, 2008 ▶); program(s) used to refine structure: *SHELXL97* (Sheldrick, 2008 ▶); molecular graphics: *SHELXTL* (Sheldrick, 2008 ▶); software used to prepare material for publication: *SHELXTL*.

## Supplementary Material

Crystal structure: contains datablocks global, I. DOI: 10.1107/S1600536810052268/rz2538sup1.cif

Structure factors: contains datablocks I. DOI: 10.1107/S1600536810052268/rz2538Isup2.hkl

Additional supplementary materials:  crystallographic information; 3D view; checkCIF report

## Figures and Tables

**Table 1:** Hydrogen-bond geometry (Å, °)

*D*—H⋯*A*	*D*—H	H⋯*A*	*D*⋯*A*	*D*—H⋯*A*
N3—H3*A*⋯O3^i^	0.90 (1)	2.04 (3)	2.928 (3)	168 (3)

Symmetry code: (i) 
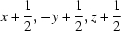
.

**Fig. 1.**
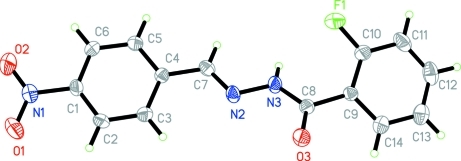
The molecular structure of the title compound, showing 30% probability displacement ellipsoids for the non-hydrogen atoms.

**Fig. 2.**
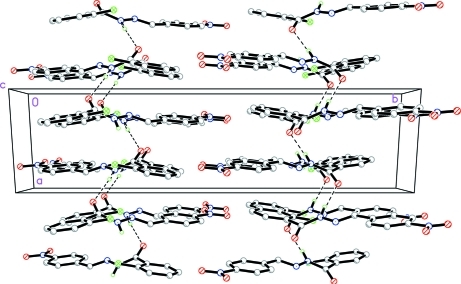
Packing diagram of the title compound, viewed along the c axis. Hydrogen bonds are shown as dashed lines.

## supplementary crystallographic information

### Comment

Hydrazone compounds derived from the condensation of aldehydes with hydrazides have been demonstrated to possess excellent biological activities (Zhong *et al.*, 2007; Raj *et al.*, 2007; Jimenez-Pulido *et al.*, 2008). Due to the easy synthesis of such compounds, a large number of hydrazone compounds have been synthesized and structurally characterized (Yehye *et al.*, 2008; Fun, Patil, Jebas *et al.*, 2008; Fun, Patil, Rao *et al.*, 2008; Yang *et al.*, 2008; Ejsmont *et al.*, 2008). Recently, we have reported a few such compounds (Ban, 2010; Ban & Li, 2008*a*,*b*; Li & Ban, 2009*a*,*b*). We report here the crystal structure of the title new compound.

In the title hydrazone compound, Fig. 1, the dihedral angle between the two substituted benzene rings C1—C6 and C9—C14 is 13.7 (3)°. The molecule exists in a *trans* configuration with respect to the central methylidene unit.

In the crystal structure, molecules are linked through intermolecular N—H···O hydrogen bonds (Table 1), forming chains along the *a* axis (Fig. 2).

### Experimental

The title compound was prepared by refluxing 4-nitrobenzaldehyde (1.0 mol) with 2-fluorobenzohydrazide (1.0 mol) in methanol (100 ml). Excess methanol was removed from the mixture by distillation. A colourless solid product was filtered, and washed three times with methanol. Colourless block-shaped crystals of the title compound were obtained from a methanol solution by slow evaporation in air.

### Refinement

Atom H3A was located in a difference Fourier map and refined isotropically, with the N—H distance restrained to 0.90 (1) Å. The remaining H atoms were placed in calculated positions (C—H = 0.93 Å) and refined as riding with *U*_iso_(H) = 1.2*U*_eq_(C).

### Crystal data

**Table:**

C_14_H_10_FN_3_O_3_	*F*(000) = 592
*M**_r_* = 287.25	*D*_x_ = 1.468 Mg m^−^^3^
Monoclinic, *P*2_1_/*n*	Mo *K*α radiation, λ = 0.71073 Å
Hall symbol: -P 2yn	Cell parameters from 680 reflections
*a* = 7.077 (2) Å	θ = 2.5–24.5°
*b* = 25.718 (4) Å	µ = 0.12 mm^−^^1^
*c* = 7.6844 (17) Å	*T* = 298 K
β = 111.640 (3)°	Block, colourless
*V* = 1300.1 (5) Å^3^	0.17 × 0.15 × 0.15 mm
*Z* = 4	

### Data collection

**Table:**

Bruker SMART CCD area-detector diffractometer	2810 independent reflections
Radiation source: fine-focus sealed tube	1155 reflections with *I* > 2σ(*I*)
graphite	*R*_int_ = 0.074
ω scans	θ_max_ = 27.0°, θ_min_ = 3.0°
Absorption correction: multi-scan (*SADABS*; Sheldrick, 1996)	*h* = −9→8
*T*_min_ = 0.981, *T*_max_ = 0.983	*k* = −32→27
6999 measured reflections	*l* = −5→9

### Refinement

**Table:**

Refinement on *F*^2^	Primary atom site location: structure-invariant direct methods
Least-squares matrix: full	Secondary atom site location: difference Fourier map
*R*[*F*^2^ > 2σ(*F*^2^)] = 0.067	Hydrogen site location: inferred from neighbouring sites
*wR*(*F*^2^) = 0.173	H atoms treated by a mixture of independent and constrained refinement
*S* = 0.98	*w* = 1/[σ^2^(*F*_o_^2^) + (0.0571*P*)^2^] where *P* = (*F*_o_^2^ + 2*F*_c_^2^)/3
2810 reflections	(Δ/σ)_max_ < 0.001
193 parameters	Δρ_max_ = 0.25 e Å^−^^3^
1 restraint	Δρ_min_ = −0.22 e Å^−^^3^

### Special details

**Table:**

Geometry. All e.s.d.'s (except the e.s.d. in the dihedral angle between two l.s. planes) are estimated using the full covariance matrix. The cell e.s.d.'s are taken into account individually in the estimation of e.s.d.'s in distances, angles and torsion angles; correlations between e.s.d.'s in cell parameters are only used when they are defined by crystal symmetry. An approximate (isotropic) treatment of cell e.s.d.'s is used for estimating e.s.d.'s involving l.s. planes.
Refinement. Refinement of *F*^2^ against ALL reflections. The weighted *R*-factor *wR* and goodness of fit *S* are based on *F*^2^, conventional *R*-factors *R* are based on *F*, with *F* set to zero for negative *F*^2^. The threshold expression of *F*^2^ > σ(*F*^2^) is used only for calculating *R*-factors(gt) *etc*. and is not relevant to the choice of reflections for refinement. *R*-factors based on *F*^2^ are statistically about twice as large as those based on *F*, and *R*- factors based on ALL data will be even larger.

### Fractional atomic coordinates and isotropic or equivalent isotropic displacement parameters (Å^2^)

**Table:**

	*x*	*y*	*z*	*U*_iso_*/*U*_eq_	
F1	0.2225 (4)	0.24829 (9)	0.4967 (3)	0.0822 (8)	
N1	0.2495 (5)	0.48648 (15)	−0.5057 (5)	0.0631 (10)	
N2	0.2488 (4)	0.29033 (11)	0.0113 (4)	0.0432 (8)	
N3	0.2828 (5)	0.25789 (11)	0.1631 (4)	0.0464 (8)	
O1	0.2201 (5)	0.47230 (12)	−0.6636 (4)	0.0978 (12)	
O2	0.2646 (6)	0.53203 (12)	−0.4634 (5)	0.1069 (13)	
O3	0.0937 (4)	0.19271 (9)	−0.0144 (3)	0.0547 (8)	
C1	0.2637 (5)	0.44701 (14)	−0.3635 (5)	0.0438 (9)	
C2	0.2406 (6)	0.39574 (14)	−0.4128 (5)	0.0486 (10)	
H2	0.2183	0.3855	−0.5348	0.058*	
C3	0.2511 (5)	0.35963 (14)	−0.2776 (5)	0.0465 (10)	
H3	0.2325	0.3246	−0.3094	0.056*	
C4	0.2891 (5)	0.37471 (13)	−0.0952 (5)	0.0374 (8)	
C5	0.3140 (5)	0.42716 (14)	−0.0505 (5)	0.0474 (10)	
H5	0.3419	0.4376	0.0723	0.057*	
C6	0.2981 (5)	0.46400 (13)	−0.1853 (5)	0.0494 (10)	
H6	0.3103	0.4993	−0.1564	0.059*	
C7	0.3085 (5)	0.33691 (14)	0.0518 (5)	0.0448 (10)	
H7	0.3652	0.3470	0.1766	0.054*	
C8	0.2013 (5)	0.21010 (13)	0.1394 (5)	0.0404 (9)	
C9	0.2475 (5)	0.17828 (13)	0.3115 (5)	0.0361 (8)	
C10	0.2603 (5)	0.19735 (14)	0.4817 (5)	0.0484 (10)	
C11	0.3020 (6)	0.16713 (18)	0.6393 (5)	0.0627 (12)	
H11	0.3100	0.1815	0.7529	0.075*	
C12	0.3310 (6)	0.11534 (18)	0.6222 (6)	0.0658 (12)	
H12	0.3597	0.0940	0.7266	0.079*	
C13	0.3190 (5)	0.09383 (15)	0.4558 (6)	0.0595 (11)	
H13	0.3399	0.0584	0.4478	0.071*	
C14	0.2757 (5)	0.12515 (13)	0.2996 (6)	0.0489 (10)	
H14	0.2653	0.1105	0.1858	0.059*	
H3A	0.374 (4)	0.2695 (13)	0.272 (3)	0.073*	

### Atomic displacement parameters (Å^2^)

**Table:**

	*U*^11^	*U*^22^	*U*^33^	*U*^12^	*U*^13^	*U*^23^
F1	0.130 (2)	0.0599 (16)	0.0721 (18)	−0.0116 (15)	0.0550 (17)	−0.0204 (13)
N1	0.084 (3)	0.055 (2)	0.051 (2)	0.003 (2)	0.026 (2)	0.009 (2)
N2	0.0457 (18)	0.0456 (19)	0.0314 (18)	−0.0036 (15)	0.0062 (15)	0.0066 (15)
N3	0.058 (2)	0.0411 (19)	0.0300 (17)	−0.0090 (16)	0.0044 (15)	0.0041 (16)
O1	0.163 (3)	0.085 (2)	0.053 (2)	−0.007 (2)	0.048 (2)	0.0113 (18)
O2	0.190 (4)	0.051 (2)	0.084 (3)	0.003 (2)	0.054 (2)	0.0158 (19)
O3	0.0665 (18)	0.0531 (17)	0.0306 (15)	−0.0094 (14)	0.0017 (13)	−0.0006 (13)
C1	0.049 (2)	0.043 (2)	0.039 (2)	0.0089 (18)	0.0158 (19)	0.0104 (19)
C2	0.060 (3)	0.051 (3)	0.032 (2)	0.002 (2)	0.0141 (19)	−0.0050 (19)
C3	0.055 (2)	0.041 (2)	0.040 (2)	0.0008 (18)	0.014 (2)	−0.0036 (19)
C4	0.035 (2)	0.038 (2)	0.036 (2)	0.0016 (16)	0.0090 (17)	0.0017 (17)
C5	0.062 (3)	0.041 (2)	0.036 (2)	−0.0033 (19)	0.015 (2)	−0.0045 (18)
C6	0.065 (3)	0.034 (2)	0.050 (3)	0.0008 (19)	0.023 (2)	−0.0022 (19)
C7	0.045 (2)	0.054 (2)	0.033 (2)	−0.0012 (19)	0.0104 (18)	−0.0002 (19)
C8	0.045 (2)	0.040 (2)	0.033 (2)	0.0036 (18)	0.0109 (18)	0.0010 (18)
C9	0.037 (2)	0.039 (2)	0.031 (2)	−0.0037 (16)	0.0101 (16)	−0.0021 (16)
C10	0.056 (2)	0.043 (2)	0.047 (3)	−0.010 (2)	0.020 (2)	−0.010 (2)
C11	0.070 (3)	0.082 (3)	0.035 (2)	−0.023 (3)	0.018 (2)	−0.003 (2)
C12	0.058 (3)	0.076 (3)	0.054 (3)	−0.008 (2)	0.009 (2)	0.025 (3)
C13	0.053 (3)	0.053 (3)	0.070 (3)	0.003 (2)	0.020 (2)	0.015 (2)
C14	0.043 (2)	0.046 (3)	0.056 (3)	0.0008 (18)	0.016 (2)	0.004 (2)

### Geometric parameters (Å, °)

**Table:**

F1—C10	1.350 (4)	C4—C7	1.458 (5)
N1—O2	1.210 (4)	C5—C6	1.377 (5)
N1—O1	1.210 (4)	C5—H5	0.9300
N1—C1	1.468 (5)	C6—H6	0.9300
N2—C7	1.270 (4)	C7—H7	0.9300
N2—N3	1.381 (4)	C8—C9	1.485 (4)
N3—C8	1.341 (4)	C9—C10	1.368 (4)
N3—H3A	0.900 (10)	C9—C14	1.389 (4)
O3—C8	1.230 (4)	C10—C11	1.376 (5)
C1—C2	1.365 (4)	C11—C12	1.361 (5)
C1—C6	1.370 (4)	C11—H11	0.9300
C2—C3	1.375 (5)	C12—C13	1.367 (5)
C2—H2	0.9300	C12—H12	0.9300
C3—C4	1.382 (4)	C13—C14	1.383 (5)
C3—H3	0.9300	C13—H13	0.9300
C4—C5	1.387 (4)	C14—H14	0.9300
			
O2—N1—O1	121.8 (4)	N2—C7—C4	120.8 (3)
O2—N1—C1	119.7 (4)	N2—C7—H7	119.6
O1—N1—C1	118.5 (4)	C4—C7—H7	119.6
C7—N2—N3	115.1 (3)	O3—C8—N3	123.1 (3)
C8—N3—N2	120.4 (3)	O3—C8—C9	120.7 (3)
C8—N3—H3A	124 (2)	N3—C8—C9	116.2 (3)
N2—N3—H3A	115 (2)	C10—C9—C14	117.1 (3)
C2—C1—C6	123.0 (3)	C10—C9—C8	124.7 (3)
C2—C1—N1	119.6 (3)	C14—C9—C8	118.3 (3)
C6—C1—N1	117.5 (3)	F1—C10—C9	118.9 (3)
C1—C2—C3	118.4 (3)	F1—C10—C11	117.3 (4)
C1—C2—H2	120.8	C9—C10—C11	123.8 (4)
C3—C2—H2	120.8	C12—C11—C10	117.3 (4)
C2—C3—C4	120.8 (3)	C12—C11—H11	121.3
C2—C3—H3	119.6	C10—C11—H11	121.3
C4—C3—H3	119.6	C11—C12—C13	121.8 (4)
C3—C4—C5	118.9 (3)	C11—C12—H12	119.1
C3—C4—C7	121.7 (3)	C13—C12—H12	119.1
C5—C4—C7	119.3 (3)	C12—C13—C14	119.6 (4)
C6—C5—C4	121.0 (3)	C12—C13—H13	120.2
C6—C5—H5	119.5	C14—C13—H13	120.2
C4—C5—H5	119.5	C13—C14—C9	120.5 (4)
C1—C6—C5	117.8 (3)	C13—C14—H14	119.8
C1—C6—H6	121.1	C9—C14—H14	119.8
C5—C6—H6	121.1		

### Hydrogen-bond geometry (Å, °)

**Table:**

*D*—H···*A*	*D*—H	H···*A*	*D*···*A*	*D*—H···*A*
N3—H3A···O3^i^	0.90 (1)	2.04 (3)	2.928 (3)	168 (3)

Symmetry codes: (i) *x*+1/2, −*y*+1/2, *z*+1/2.
